# Histopathology Versus Imaging: A Comparative Study of Liver Biopsy, Ultrasonography, and Triphasic Computed Tomography in Paediatric Patients With Chronic Liver Disease

**DOI:** 10.7759/cureus.116694

**Published:** 2026-09-21

**Authors:** Filza Karim, Faraz Hussain, Heebah Shabbir, Sobia Naureen, Iqtadar Seerat

**Affiliations:** 1 Department of Gastroenterology and Hepatology, Pakistan Kidney and Liver Institute and Research Center, Lahore, PAK; 2 Department of Paediatric and Child Health, Pakistan Kidney and Liver Institute and Research Center, Lahore, PAK; 3 Department of Paediatric Gastroenterology and Hepatology, Pakistan Kidney and Liver Institute and Research Center, Lahore, PAK

**Keywords:** chronic liver disease, histopathology, liver biopsy, paediatric, triphasic computed tomography

## Abstract

Background: Assessment of liver fibrosis and cirrhotic changes in children with chronic liver disease (CLD) can be challenging, particularly when invasive assessment is limited by clinical factors. This study compared ultrasonography and triphasic computed tomography (CT) findings with histopathology in paediatric patients with CLD.

Aim: The aim of the study is to compare ultrasonography and triphasic CT findings with liver biopsy findings in paediatric patients with CLD and to assess their concordance with histopathology using predefined study-specific categories of good, partial, and poor agreement.

Materials and methods: A prospective analytical observational study was conducted at the Department of Paediatrics and Paediatric Gastroenterology, Pakistan Kidney and Liver Institute and Research Center, Lahore, from April 10, 2026 to July 10, 2026. A total of 100 children aged 0-15 years with CLD or decompensated CLD were included. Clinical, biochemical, ultrasonographic, triphasic CT, and liver biopsy findings were recorded. Histopathology was used as the reference standard. Agreement analysis was performed in patients with adequate biopsy samples using predefined study-specific criteria. Paired binary comparisons were assessed using the McNemar test, while the Bowker test was used for comparison across the three agreement categories. A p-value of less than 0.05 was considered statistically significant.

Results: Liver biopsy was performed in 93 patients, with adequate biopsy samples obtained in 90 patients. Among patients with adequate biopsy samples, 81/90 (90%) had F3 or F4 fibrosis. Good agreement with histopathology was observed more frequently with triphasic CT than with ultrasonography (59/90 versus 17/90, p < 0.001). Good or partial agreement was also higher with triphasic CT than with ultrasonography (83/90 versus 61/90, p < 0.001).

Conclusion: Triphasic CT demonstrated greater concordance with histopathology than ultrasonography in this predominantly advanced CLD cohort. These findings mainly reflect the assessment of advanced fibrosis and cirrhotic changes and should not be extrapolated to early-stage disease. Triphasic CT may provide complementary structural information, while liver biopsy remains necessary when definitive histological diagnosis or fibrosis staging is required.

## Introduction

Chronic liver disease (CLD) in children is a progressive condition characterised by persistent liver injury, fibrosis, architectural distortion, and, in advanced cases, cirrhosis. The causes of CLD in children differ from those in adults and include cholestatic, autoimmune, metabolic, hereditary, and vascular disorders [1]. Paediatric cirrhosis is uncommon, but its clinical burden is important. A population-based study from Ontario reported that the incidence of cirrhosis in children increased almost fourfold over two decades [2]. Early recognition is therefore important because timely diagnosis can guide treatment, help prevent complications, and identify children who may require referral for liver transplantation.

Liver biopsy remains the reference standard for confirming the diagnosis and staging fibrosis in CLD. It allows direct histopathological assessment of fibrosis, inflammation, cirrhosis, and disease-specific tissue changes. A recent paediatric liver biopsy study from Pakistan showed that biopsy helped confirm or support the diagnosis in children with unexplained liver disease and in those whose ultrasound or computed tomography (CT) findings required tissue confirmation [3].

Biopsy, however, has important limitations. It is invasive and may not be safe in children with a deranged international normalised ratio (INR), severe thrombocytopenia, gross ascites, or clinical instability. Sampling error and inadequate tissue can also affect interpretation. For these reasons, non-invasive imaging has become an important component of the diagnostic pathway in paediatric CLD. Ultrasonography (USG) is usually the first-line imaging investigation because it is widely available, safe, and repeatable. It can assess liver size, surface characteristics, parenchymal echotexture, splenomegaly, ascites, and features of portal hypertension. A recent review of paediatric cirrhosis highlighted that USG is commonly used as the primary imaging modality, while CT and magnetic resonance imaging are used when more detailed anatomical assessment is required [4].

Triphasic CT provides additional structural and vascular information in selected children. It can demonstrate cirrhotic parenchymal changes, nodular liver contour, segmental volume alterations, vascular abnormalities, portosystemic collaterals, ascites, and focal liver lesions. Paediatric liver imaging guidance also supports the selective use of contrast-enhanced CT when detailed anatomical assessment is required or when magnetic resonance imaging is unsuitable [4].

Recent literature has increasingly focused on non-invasive assessment of hepatic fibrosis. A systematic review of the paediatric population reported that ultrasound elastography has potential for fibrosis assessment, although liver biopsy continues to serve as the reference standard in many clinical settings [5]. A systematic review supporting the American Association for the Study of Liver Diseases practice guideline also demonstrated that imaging-based non-invasive liver disease assessment can provide useful information for fibrosis staging, particularly in advanced disease [6]. However, much of the available evidence has focused on elastography or other specialised non-invasive techniques rather than direct comparison of routine USG and triphasic CT with histopathology.

A recent study from Pakistan compared shear-wave elastography with liver biopsy in children with CLD but did not directly compare routine USG and triphasic CT with histopathological findings [7]. Consequently, evidence regarding how routine USG and triphasic CT correspond with histopathological findings in paediatric CLD remains limited. This question is clinically relevant because children with advanced CLD may have coagulopathy, thrombocytopenia, ascites, or clinical instability that can complicate invasive assessment.

The present study was planned with the hypothesis that triphasic CT would demonstrate greater agreement with histopathological findings than routine USG, particularly in children with advanced fibrosis and cirrhotic changes. The objective was to compare USG and triphasic CT findings with liver biopsy findings in paediatric patients with CLD and to evaluate their concordance with histopathology using predefined study-specific categories of good, partial, and poor agreement. These categories were intended to assess morphological agreement with histopathology rather than formal diagnostic accuracy or exact radiological staging of fibrosis.

## Materials and methods

A prospective analytical observational study was conducted at the Department of Paediatrics and Paediatric Gastroenterology, Pakistan Kidney and Liver Institute and Research Center, Lahore, from April 10, 2026 to July 10, 2026. The study included paediatric patients with CLD or decompensated chronic liver disease (DCLD) who underwent clinical, biochemical, radiological, and histopathological assessment as part of their diagnostic work-up. Ethical approval was obtained from the Institutional Review Board (IRB) of Pakistan Kidney and Liver Institute and Research Center, Lahore. The IRB number was 00172026, and the approval reference was PKLI/IRB/AP/00172026. Full quorum approval was granted on 09 April 2026. Written informed consent was obtained from parents or legal guardians, and assent was obtained from children where age-appropriate. The sample size was calculated using the single population proportion formula. The calculation was based on the expected proportion of biopsy-confirmed diagnoses reported in a recent paediatric liver biopsy study from Pakistan [3]. The reported proportion was 82.6%. Using this proportion, a 95% confidence level and an 8% margin of error, the calculated sample size was 86 patients. After adding a 10% allowance for incomplete imaging, inadequate biopsy samples, missing reports, or patients in whom biopsy could not be safely completed, the minimum required sample size was 95 patients. The final sample size was rounded to 100 patients. The sample size was intended primarily to describe the pattern of agreement between imaging and histopathology. Because reliable prior data for the paired difference in agreement between USG and triphasic CT were not available at the time of study planning, the study was not specifically powered for the paired comparison between the two imaging modalities.

Children aged 0-15 years with CLD or DCLD were included. CLD was diagnosed on the basis of clinical features, biochemical abnormalities, and radiological findings. Patients were enrolled after assessment by the paediatric gastroenterology team. All enrolled patients underwent abdominal USG and triphasic CT as part of their radiological assessment. Liver biopsy was performed when clinically indicated and considered safe. Patients with adequate biopsy samples were included in the primary agreement analysis. Patients in whom biopsy was not performed because of clinical contraindications were analysed descriptively and excluded from the primary agreement analysis. Patients with inadequate biopsy samples were also excluded from the primary agreement analysis. For patients who underwent biopsy, imaging was included only when it was performed within four weeks before or after liver biopsy. Patients with acute liver failure or acute hepatitis without underlying CLD were excluded. Patients with previous liver transplantation or hepatic resection were also excluded. Patients with known hepatic malignancy, focal infiltrative liver disease, or poor-quality imaging were not included. Patients with contraindications to triphasic CT, including severe renal impairment or known iodinated contrast allergy, were not included if triphasic CT could not be performed. Patients with an interval of more than four weeks between imaging and biopsy were excluded from the primary agreement analysis.

Demographic details, clinical diagnosis, disease duration, and presenting features were recorded. Clinical features included jaundice, abdominal distension, pruritus, failure to thrive, gastrointestinal bleeding, hepatomegaly, splenomegaly, ascites, and encephalopathy. Laboratory variables included total bilirubin, direct bilirubin, alanine aminotransferase, aspartate aminotransferase, alkaline phosphatase, gamma-glutamyl transferase, serum albumin, INR, and platelet count. All USG examinations were performed or reviewed by the same experienced paediatric radiologist. The liver was assessed for size, surface margin, parenchymal echotexture, nodularity, and focal lesions. The spleen was assessed for splenomegaly. Ascites, portal vein dilatation, and other features of portal hypertension were also recorded. The radiologist was blinded to the histopathological findings at the time of imaging assessment. USG findings were subsequently compared with histopathological findings in patients with adequate biopsy samples.

Triphasic CT was performed according to the institutional paediatric radiology protocol using multidetector CT acquisition. A non-ionic, low-osmolality iodinated contrast agent was administered intravenously at a dose of 2 mL/kg body weight, without exceeding the usual institutional adult maximum dose. Contrast was administered using a power injector, with the injection rate adjusted according to the child's body size and intravenous cannula calibre. The late arterial phase was acquired approximately 16-20 seconds after initiation of contrast administration, the portal venous phase at approximately 55-60 seconds, and the delayed phase at approximately 120-180 seconds. Bolus tracking could be used for the late arterial acquisition when required. The arterial and delayed acquisitions were restricted to the liver and upper abdomen, while the portal venous acquisition provided the required abdominal coverage. The CT technique was adjusted for patient size. A tube voltage of approximately 80-100 kVp was used in most children, with up to 120 kVp used in larger adolescents when necessary for adequate penetration. Automatic tube-current modulation was used rather than a fixed tube current to adapt radiation output to body size. Gantry rotation time was approximately 0.5 seconds, with a pitch of approximately 0.9-1.0. Images were reconstructed using a standard soft-tissue algorithm, with routine section thickness of approximately 3-5 mm and additional thin-section reconstructions of approximately 0.5-1.5 mm when required for detailed assessment. Radiation exposure was minimised by adjusting tube voltage and tube current to patient size, restricting scan coverage to the clinically required anatomical region, and avoiding an additional unenhanced acquisition. Multiphase imaging was performed according to the clinical liver protocol, with dose optimisation following the as-low-as-reasonably-achievable principle. Sedation was not routinely required because of the short CT acquisition time. It was considered only in younger or uncooperative children who were unable to remain still or comply adequately with breathing instructions and, when required, was administered according to the institutional paediatric sedation protocol. The liver was assessed for cirrhotic parenchymal changes, nodular contour, segmental volume changes, enhancement pattern, vascular abnormalities, portosystemic collaterals, ascites, and focal lesions. Triphasic CT examinations were reviewed by the same experienced paediatric radiologist who reviewed the USG examinations. The radiologist was blinded to histopathological findings during imaging assessment. Because the same radiologist evaluated both imaging modalities, interobserver agreement between radiologists was not assessed.

Liver biopsy was performed when clinically indicated and safe. All biopsies were performed by the percutaneous approach under institutional protocol. Biopsy specimens were assessed by the histopathology department. Fibrosis stage, inflammation, bridging fibrosis, cirrhosis, steatosis, and malignancy were recorded. Fibrosis was recorded on an F0-F4 scale according to the institutional histopathology reporting system. Histopathological specimens were evaluated by a pathologist who was blinded to the imaging-based agreement classification. For the primary analysis, histopathological fibrosis was grouped into three categories: F0-F2 was classified as non-advanced fibrosis, F3 as advanced fibrosis, and F4 as cirrhosis. Histopathology was considered the reference standard. Agreement analysis was performed only in patients with adequate biopsy samples. Patients in whom biopsy was not performed or in whom biopsy tissue was inadequate were excluded from the primary agreement analysis. USG and triphasic CT findings were matched separately with the corresponding histopathological categories using predefined study-specific criteria. Imaging categories were assigned on the basis of the overall morphological pattern rather than a numerical radiological fibrosis score.

Non-advanced disease on imaging was characterised by the absence of definite cirrhotic morphology and the absence of convincing radiological evidence of portal hypertension. Advanced disease was characterised by morphological features of CLD or portal hypertension without definite cirrhotic morphology. Cirrhosis on imaging was characterised by definite cirrhotic morphology, including nodular liver contour or marked architectural alteration, with or without associated features of portal hypertension such as splenomegaly, ascites, portal vein dilatation, or portosystemic collaterals. Good agreement was defined as imaging findings corresponding to the histopathological category. Thus, good agreement included non-advanced imaging findings with F0-F2 histopathology, imaging features of advanced CLD without definite cirrhosis with F3 histopathology, and definite cirrhotic imaging morphology with F4 histopathology. Partial agreement was defined as imaging findings that supported CLD or portal hypertension but did not fully correspond to the biopsy-based fibrosis category. This included cases in which imaging demonstrated CLD but could not reliably distinguish advanced fibrosis from cirrhosis. Poor agreement was defined as major discordance between imaging and histopathology, including normal or non-specific imaging in patients with advanced fibrosis or cirrhosis, or imaging findings suggesting advanced disease when histopathology showed non-advanced fibrosis. These good, partial, and poor agreement categories were predefined study-specific criteria intended to assess morphological concordance between imaging and histopathology. They were not intended to represent a validated radiological fibrosis staging system or formal measures of diagnostic accuracy.

## Results

Baseline clinical and biochemical characteristics

There were 100 paediatric patients in the study. The mean age was 7.50 ± 3.59 years. There were 55 male patients and 45 female patients. Most patients had a disease duration of more than 12 months. Jaundice and hepatomegaly were present in all patients. Splenomegaly was present in most patients. Progressive familial intrahepatic cholestasis (PFIC) was the most common diagnosis, followed by autoimmune hepatitis and Wilson disease. Baseline demographic, clinical, and biochemical characteristics are shown in Table 1.

**Table 1:** Baseline demographic, clinical, and biochemical characteristics of the study population Continuous variables are presented as mean ± standard deviation. Categorical variables are presented as n (%).

Variable	Value
Total patients	100
Demographic characteristics	
Age, years	7.50 ± 3.59
Weight, kg	19.11 ± 8.79
Height, cm	112.18 ± 17.86
Male sex	55 (55%)
Female sex	45 (45%)
Duration of disease	
< 6 months	10 (10%)
6–12 months	18 (18%)
> 12 months	72 (72%)
Clinical diagnosis	
Progressive familial intrahepatic cholestasis	38 (38%)
Autoimmune hepatitis	22 (22%)
Wilson disease	15 (15%)
Biliary atresia – failed Kasai	10 (10%)
Cryptogenic chronic liver disease	10 (10%)
Primary sclerosing cholangitis	5 (5%)
Clinical features	
Jaundice	100 (100%)
Abdominal distension	46 (46%)
Increased sleepiness	16 (16%)
Gastrointestinal bleeding	10 (10%)
Failure to thrive	52 (52%)
Pruritus	70 (70%)
Hepatomegaly	100 (100%)
Splenomegaly	85 (85%)
Ascites	37 (37%)
Encephalopathy	9 (9%)
Biochemical characteristics	
Total bilirubin	14.04 ± 7.25
Direct bilirubin	11.12 ± 6.49
Alanine aminotransferase, IU/L	493.93 ± 303.12
Aspartate aminotransferase, IU/L	613.27 ± 403.52
Alkaline phosphatase, IU/L	707.91 ± 368.28
Gamma-glutamyl transferase, IU/L	301.67 ± 258.26
Serum albumin, g/dL	2.58 ± 0.55
International normalized ratio	1.86 ± 0.59
Platelet count	106.66 ± 46.86

Histopathological findings

Liver biopsy was performed in 93 patients, and all biopsies were performed through the percutaneous approach. Biopsy was not performed in seven patients due to clinical contraindications. Adequate biopsy tissue was obtained in 90 patients, while three samples were inadequate for fibrosis staging. Advanced fibrosis was the dominant histopathological finding. Among patients with adequate biopsy samples, 81/90 (90%) had stage F3 or F4 fibrosis. Stage F4 fibrosis was the most frequent stage. Histopathological findings and fibrosis stage distribution are shown in Table 2.

**Table 2:** Histopathological findings and fibrosis stage distribution Data are presented as n (%) unless stated otherwise. Biopsy-related complication percentages were calculated using the 93 patients who underwent percutaneous liver biopsy as the denominator.

Variable	Value
Total patients	100
Biopsy type	
Percutaneous biopsy	93 (93%)
Not performed	7 (7%)
Biopsy sample adequacy	
Adequate	90 (90%)
Inadequate	3 (3%)
Not performed	7 (7%)
Fibrosis stage	
F0-F1	2 (2%)
F2	7 (7%)
F3	33 (33%)
F4	48 (48%)
Inadequate sample	3 (3%)
Not assessed	7 (7%)
Advanced fibrosis/cirrhosis among adequate biopsy cases	81/90 (90%)
Histological steatosis	
Yes	6 (6%)
No	84 (84%)
Not assessed	10 (10%)
Histological inflammation	
Mild	5 (5%)
Moderate	56 (56%)
Severe	29 (29%)
Not assessed	10 (10%)
Bridging fibrosis	
Yes	86 (86%)
No	4 (4%)
Not assessed	10 (10%)
Histological cirrhosis	
Yes	48 (48%)
No	42 (42%)
Not assessed	10 (10%)
Histological malignancy	
No	90 (90%)
Not assessed	10 (10%)
Biopsy complications among 93 biopsied patients	
No complication	88/93 (94.62%)
Any complication	5/93 (5.38%)
Transient observation delay after biopsy	2/93 (2.15%)
Small self-limited subcapsular haematoma	1/93 (1.08%)
Mild post-biopsy pain managed conservatively	1/93 (1.08%)
Minor bleeding risk/poor sample adequacy noted	1/93 (1.08%)

Diagnostic agreement between imaging and histopathology

Agreement analysis was performed in 90 patients with adequate biopsy samples. No patient with an adequate biopsy sample was excluded from the triphasic CT comparison because of a CT contraindication or missing CT assessment; therefore, the same 90 patients were included in both USG and triphasic CT agreement analyses. Triphasic CT showed better agreement with histopathology than USG. Good agreement was observed in 59 patients on triphasic CT, compared with 17 patients on USG. Poor agreement was lower with triphasic CT. The combined good or partial agreement was also higher for triphasic CT than USG (92.22% versus 67.78%, p < 0.001). These findings are shown in Table 3.

**Table 3:** Diagnostic agreement of ultrasonography and triphasic computed tomography with histopathology Agreement analysis was performed only in patients with adequate biopsy samples. Ten patients were excluded because biopsy was either not performed or the biopsy sample was inadequate. The McNemar test was used for paired binary comparisons. The Bowker test was used for the paired three-category agreement comparison. The paired agreement distribution used for these tests is provided in the table in Appendices. No p-value was calculated for total, included, or excluded patients because these rows describe the analysis denominator only.

Agreement parameter	Ultrasonography	Triphasic computed tomography	Statistical test	Test statistic	df	p-value
Total patients	100	100	Not applicable	—	—	—
Not included in agreement analysis	10 (10.00%)	10 (10.00%)	Not applicable	—	—	—
Patients included in agreement analysis	90 (90.00%)	90 (90.00%)	Not applicable	—	—	—
Overall agreement category			Bowker test	χ² = 39.36	3	<0.001
Good agreement	17 (18.89%)	59 (65.56%)	—	—	—	—
Partial agreement	44 (48.89%)	24 (26.67%)	—	—	—	—
Poor agreement	29 (32.22%)	7 (7.78%)	—	—	—	—
Paired binary agreement comparisons						
Good agreement versus not good agreement	17 (18.89%)	59 (65.56%)	McNemar test	χ² = 33.92	1	<0.001
Good or partial agreement versus poor agreement	61 (67.78%)	83 (92.22%)	McNemar test	χ² = 17.29	1	<0.001

## Discussion

PFIC was the most common diagnosis in the present study. Autoimmune hepatitis, Wilson disease, biliary atresia with failed Kasai procedure, cryptogenic CLD, and primary sclerosing cholangitis were also identified. The predominance of cholestatic and hereditary liver diseases is expected in a paediatric liver referral centre. A Pakistani study on genetic cholestatic diseases also reported a broad range of inherited cholestatic disorders in children, including PFIC-related mutations [8]. The disease distribution in the present study therefore reflects the expected pattern of paediatric CLD in a specialised hepatology setting.

PFIC formed the largest diagnostic group in this cohort. PFIC commonly presents with jaundice, pruritus, hepatomegaly, cholestasis, and progressive liver injury during childhood. A Pakistani study on PFIC type 3 also reported jaundice, pruritus, neonatal cholestasis, and raised gamma-glutamyl transferase as important clinical features in affected children [9]. The high proportion of PFIC in the present study is therefore clinically plausible and supports the need for early assessment of liver damage in these patients.

Advanced fibrosis was common in the present cohort. Persistent cholestasis can produce ongoing hepatocellular injury, fibrosis, portal hypertension, and cirrhosis. A recent study on PFIC outcomes showed that disease progression and response to biliary diversion differ according to genetic subtype, and some children still progress towards liver transplantation despite intervention [10]. PFIC should therefore be considered an important contributor to advanced fibrosis in paediatric CLD.

Autoimmune hepatitis was the second most common diagnosis in the present study. The disease can progress to advanced fibrosis if diagnosis or treatment is delayed. A regional paediatric study from Jordan showed that autoimmune hepatitis in children often requires histological assessment and long-term treatment monitoring [11]. The presence of autoimmune hepatitis in the present cohort supports the role of liver biopsy in confirming inflammation, interface activity, and fibrosis severity.

Some children with CLD may have overlapping clinical, biochemical, immunological, or histological features. A recent paediatric study on autoimmune hepatitis-like liver disease showed that different liver diseases may present with similar biochemical and histological patterns [12]. The present findings support careful interpretation of imaging along with clinical, laboratory, and histopathological data, especially when the diagnosis is uncertain.

Wilson disease was present in 15% of patients. Wilson disease can present as chronic hepatitis, cirrhosis, or DCLD in children. A recent study showed that hepatic ultrastructural features can help distinguish paediatric Wilson disease from autoimmune hepatitis and fatty liver disease [13]. The presence of Wilson disease in the present cohort supports the value of histopathology when clinical and biochemical findings overlap with other liver diseases.

Biliary atresia with a failed Kasai procedure was present in 10% of patients. These children may develop progressive fibrosis, portal hypertension, and cirrhosis when bile drainage remains inadequate. A study on biliary atresia showed that shear-wave elastography, performed before and after Kasai portoenterostomy, was useful for predicting hepatic fibrosis and native liver outcomes [14]. The finding in the present study is therefore consistent with the known progressive nature of failed Kasai disease.

Advanced fibrosis was the dominant histopathological finding. Among patients with adequate biopsy samples, 81 of 90 had stage F3 or F4 fibrosis. The high frequency of advanced fibrosis is expected because the study included children with CLD and DCLD who were managed at a tertiary liver centre. A paediatric study comparing non-invasive elastography methods with histopathology also showed that advanced fibrosis is an important target for non-invasive assessment in children with liver disease [15]. The fibrosis distribution in the present study therefore supports the clinical severity of the study population.

USG showed lower good agreement with histopathology than triphasic CT. Routine USG relies on visible morphological findings, including altered echotexture, surface irregularity, splenomegaly, ascites, and features of portal hypertension. Early or heterogeneous fibrosis may not always produce clear sonographic changes. A paediatric study on shear-wave elastography showed that ultrasound-based stiffness assessment can improve fibrosis evaluation, but routine sonographic morphology alone may be less precise for fibrosis staging [16]. USG remains a useful first-line, accessible tool, but it should not be used alone for fibrosis staging when biopsy or advanced imaging is available.

Imaging did not match histopathology in all patients. The difference is scientifically plausible because fibrosis begins as a microscopic process before it becomes visible as gross parenchymal distortion. A study on young Fontan patients showed that elastography could both overestimate and underestimate fibrosis compared with biopsy [17]. The present findings support the same principle and show that imaging and biopsy assess different aspects of liver disease.

Triphasic CT showed stronger agreement with histopathology than USG. Good or partial agreement was seen in 83 of 90 patients with adequate biopsy samples. CT can show nodular liver contour, parenchymal heterogeneity, segmental volume changes, collaterals, ascites, and vascular abnormalities more clearly than routine USG. A paediatric study on magnetic resonance elastography also showed that advanced imaging methods can improve non-invasive assessment of fibrosis in children with CLD [18]. Triphasic CT, therefore, appears more useful than routine USG for recognising advanced cirrhotic changes in this cohort.

Histopathology remained the reference standard in the present study. Triphasic CT showed significantly better agreement with biopsy than USG. A paediatric study comparing transient elastography with imaging and biopsy also reported that non-invasive tests provide useful information, but their performance varies according to modality and disease context [19]. The present findings indicate that triphasic CT may provide complementary structural information in children with advanced CLD; however, because patients without adequate biopsy samples were excluded from the primary agreement analysis, this study does not directly establish the performance of CT in children in whom biopsy is unsafe or not feasible. Liver biopsy remains the reference standard for definitive histological diagnosis and fibrosis staging.

Non-invasive assessment is important in children because a biopsy may be difficult in the presence of coagulopathy, thrombocytopenia, ascites, or poor general condition. A study on children with chronic viral hepatitis showed that non-invasive methods can help detect fibrosis, but their performance should be assessed against biopsy when possible [20]. The present study supports a balanced approach in which imaging guides management, but biopsy is performed when clinically safe and required.

Liver biopsy was not performed in seven patients and was inadequate in three. This reflects real clinical practice because paediatric liver biopsy requires careful patient selection, especially in children with a deranged coagulation profile, ascites, or clinical instability. A recent paediatric study on ultrasound-guided percutaneous liver biopsy confirmed that biopsy is valuable for diagnosis and follow-up, but sample adequacy and procedure-related complications remain important considerations [21]. These findings illustrate the practical limitations of liver biopsy in paediatric CLD; however, the present study did not directly evaluate the diagnostic performance of imaging in patients in whom biopsy could not be performed.

Primary sclerosing cholangitis was present in a small number of patients. The low frequency is expected because paediatric primary sclerosing cholangitis is less common than PFIC, autoimmune hepatitis, Wilson disease, and biliary atresia in many liver referral settings. A recent childhood primary sclerosing cholangitis cohort showed that prognosis can vary and that long-term follow-up is required [22]. Primary sclerosing cholangitis should therefore be interpreted as part of the broader paediatric CLD spectrum rather than the dominant diagnosis in this cohort.

The main clinical message from the present study is clear. Liver biopsy remains the reference standard for diagnosing and staging paediatric CLD. Triphasic CT showed better agreement with histopathology than USG, especially in advanced fibrosis and cirrhotic changes. USG remains valuable as an initial and accessible test, particularly in resource-limited settings. Accordingly, USG should remain the first-line imaging modality because of its safety and accessibility, while triphasic CT may provide complementary structural information when clinically indicated. Its use should be individualised, particularly in children, because of ionising radiation exposure, and it should not be considered a substitute for histopathological assessment.

The study has several limitations. It was conducted at a single tertiary care centre, and the study population was heavily weighted towards advanced liver disease, with 81 of 90 patients with adequate biopsy samples having F3 or F4 fibrosis. This spectrum may have increased the observed agreement between imaging and histopathology and limits the generalisability of the findings to children with early or intermediate fibrosis. Selection bias is also possible because liver biopsy was performed only when clinically indicated and considered safe, while patients in whom biopsy could not be performed were excluded from the primary agreement analysis. The agreement categories of good, partial, and poor agreement were predefined study-specific criteria rather than a validated radiological fibrosis scoring system, which may limit reproducibility. Although imaging interpretation and histopathological assessment were performed independently and with blinding, interobserver agreement could not be assessed because the same radiologist reviewed both imaging modalities. The sample-size calculation was based on an expected biopsy-confirmed diagnostic proportion rather than the primary paired comparison between USG and triphasic CT, and the study was therefore not specifically powered for that comparison. In addition, triphasic CT involves ionising radiation, and the use of multiple contrast-enhanced phases increases radiation exposure in children. Therefore, these findings should not be interpreted as supporting routine triphasic CT for all children with CLD, particularly those with early-stage disease. The study also did not include elastography or magnetic resonance-based techniques, which limits comparison with other non-invasive imaging approaches.

Future studies should include larger multicentre paediatric cohorts. A wider distribution of fibrosis stages would help assess imaging performance separately in early and advanced disease. Future work should compare routine USG, triphasic CT, elastography, and magnetic resonance-based techniques in the same patients. Standardised radiological scoring for cirrhotic morphology, portal hypertension, and focal lesions may improve agreement between radiology and histopathology. Long-term follow-up can also show whether CT and USG findings predict outcomes such as portal hypertension, transplant referral, or progression of liver failure.

## Conclusions

Triphasic CT showed greater agreement with histopathology than USG in this predominantly advanced-CLD paediatric cohort, particularly in patients with advanced fibrosis and cirrhotic changes. These findings should not be extrapolated to early-stage disease or interpreted as evidence that CT can replace liver biopsy. Triphasic CT may provide complementary structural information when clinically indicated; however, its use in children should be individualised because of ionising radiation exposure. USG remains an important first-line imaging modality because of its safety, accessibility, and repeatability, while liver biopsy remains the reference standard when definitive histological diagnosis or fibrosis staging is required.

## Appendices

**Table 4:** Paired cross-tabulation of ultrasonography and triphasic CT agreement categories among adequate biopsy cases Statistical interpretation: The Bowker test showed a significant difference in the overall paired agreement distribution between USG and triphasic CT (χ² = 39.36, df = 3, p < 0.001). The McNemar test showed that triphasic CT had significantly higher good agreement with histopathology than USG (59/90 versus 17/90; χ² = 33.92, df = 1, p < 0.001). The combined good or partial agreement was also higher for triphasic CT than ultrasonography (83/90 versus 61/90; χ² = 17.29, df = 1, p < 0.001). USG: ultrasonography; CT: computed tomography. Analysis was performed in 90 patients with adequate biopsy samples.

Ultrasonography agreement category	CT good agreement	CT partial agreement	CT poor agreement	Total
USG good agreement	12	4	1	17
USG partial agreement	32	10	2	44
USG poor agreement	15	10	4	29
Total	59	24	7	90
